# Epigenetic targeting of autophagy for cancer: DNA and RNA methylation

**DOI:** 10.3389/fonc.2023.1290330

**Published:** 2023-12-08

**Authors:** Luobin Lin, Yuntao Zhao, Qinzhou Zheng, Jiayang Zhang, Huaqin Li, Wenmei Wu

**Affiliations:** ^1^ Guangdong Province Key Laboratory of Biotechnology Drug Candidates, School of Life Sciences and Biopharmaceuticals, Guangdong Pharmaceutical University, Guangzhou, Guangdong, China; ^2^ School of Health Sciences, Guangzhou Xinhua University, Guangzhou, Guangdong, China

**Keywords:** 6mA methylation, m6A methylation, autophagy, cancers, therapy

## Abstract

Autophagy, a crucial cellular mechanism responsible for degradation and recycling of intracellular components, is modulated by an intricate network of molecular signals. Its paradoxical involvement in oncogenesis, acting as both a tumor suppressor and promoter, has been underscored in recent studies. Central to this regulatory network are the epigenetic modifications of DNA and RNA methylation, notably the presence of N6-methyldeoxyadenosine (6mA) in genomic DNA and N6-methyladenosine (m6A) in eukaryotic mRNA. The 6mA modification in genomic DNA adds an extra dimension of epigenetic regulation, potentially impacting the transcriptional dynamics of genes linked to autophagy and, especially, cancer. Conversely, m6A modification, governed by methyltransferases and demethylases, influences mRNA stability, processing, and translation, affecting genes central to autophagic pathways. As we delve deeper into the complexities of autophagy regulation, the importance of these methylation modifications grows more evident. The interplay of 6mA, m6A, and autophagy points to a layered regulatory mechanism, illuminating cellular reactions to a range of conditions. This review delves into the nexus between DNA 6mA and RNA m6A methylation and their influence on autophagy in cancer contexts. By closely examining these epigenetic markers, we underscore their promise as therapeutic avenues, suggesting novel approaches for cancer intervention through autophagy modulation.

## 1 Introduction

6mA DNA, which signifies adenine methylation at the sixth position, has been recently spotlighted in research as a key epigenetic marker orchestrating cellular metabolism across a wide spectrum of organisms, from bacteria to mammals (1, 2). Concurrently, there is a growing intrigue surrounding the interplay between m6A RNA methylation and autophagy. A landmark study emphasizes m6A’s pivotal regulatory role in autophagy expression (3). Moreover, researchers discerned a link between type-specific HPV infections and hTERT DNA methylation in patients suffering from invasive cervical cancer, offering an initial insight into how DNA methylation might regulate autophagy (4). On a mechanistic level, 6mA DNA modulates the expression of autophagy-related genes by influencing the accessibility of transcription factors to their target gene promoters (5).

Meanwhile, m6A methylation, characterized by its dynamic reversibility, has also attracted significant attention. Orchestrated by a sophisticated interplay of “writers” or methyltransferases, “erasers” or demethylases, and “readers” or m6A-binding proteins, m6A RNA modification stands out as a key player (6–8). The relationship between autophagy and cancer is intricate and continues to be actively investigated. It plays dual roles, both promoting and inhibiting cancer. This duality stems from the involvement of diverse ATG proteins and core complexes, such as the ULK1/2 kinase core complex, the autophagy-specific class III PI3K complex, and the ATG9A trafficking system, among others (9, 10). These components intricately coordinate a spectrum of tasks within the autophagy pathway, from initiation to degradation. Reflecting on this, it becomes clear that grasping the subtle roles of autophagy in cancer is crucial for propelling therapeutic breakthroughs. Both DNA and RNA methylation are gaining heightened acknowledgment, shaping cellular reactions to assorted stressors and ailments (11). An expanding corpus of evidence endorses the indispensable role of RNA m6A in regulating autophagy, particularly in stabilizing and enhancing the translation of autophagy-related mRNAs (12). Preliminary investigations have begun to shed light on the potential connections between 6mA methylation and its precise regulation of autophagy, notably highlighting its significant relevance in the context of cancer (13).

Autophagy, an intricately orchestrated cellular process, plays a pivotal role in maintaining cellular equilibrium by selectively degrading and recycling intracellular constituents, including damaged organelles and misfolded proteins (14, 15). The dysregulation of autophagy has been implicated in a myriad of human afflictions, among them being cancer, neurodegenerative disorders, and metabolic anomalies (16–18). Significant investigations have unraveled a direct connection between 6mA methylation and the regulation of autophagy (13). Similar to DNA methylation, m6A mRNA methylation also assumes a crucial role in regulating autophagy and adipogenesis, particularly by targeting *Atg5* and *Atg7*. Remarkably, these targets, *Atg5* and *Atg7*, are recognized by the YTHDF2 protein, a known N6-methyladenosine RNA binding entity. Furthermore, the research demonstrates that a deficiency in FTO results in a decrease in white fat mass and impedes autophagy reliant on both ATG5 and ATG7 *in vivo* (19). These investigations underscore the intricate interplay between mRNA methylation, autophagy, and cancer.

Most current research distinctively explores the roles of 6mA methylation and m6A methylation in modulating gene and protein expression, particularly in the context of autophagy. Yet, a significant gap remains in understanding the synergistic effects of 6mA and m6A methylation on autophagy regulation. Such an understanding is crucial, especially when considering the complex environment of tumor cells. As the expression levels of 6mA and m6A methylation within cells are perpetually changing, it’s pivotal to discern if one form of methylation can impact the other and, in turn, influence broader expression patterns. This holds promising implications for future research.

In this review, our aim is to elucidate the essential roles of 6mA DNA and m6A RNA methylation in autophagy regulation within cancer contexts. We offer a concise overview of autophagy’s role in cancer. Additionally, we delve into the complexities of 6mA methylation and its impact on autophagy, elucidating the underlying mechanisms of 6mA methylation and the dynamic interplay between m6A and autophagy in oncological scenarios. In conclusion, we present an in-depth analysis of m6A methylation’s pivotal role in autophagy regulation, emphasizing the promise of 6mA and m6A-guided autophagy as potential therapeutic strategies in cancer management, potentially heralding novel anticancer treatments.

## 2 Overview of autophagy in cancer

**Figure 1:**
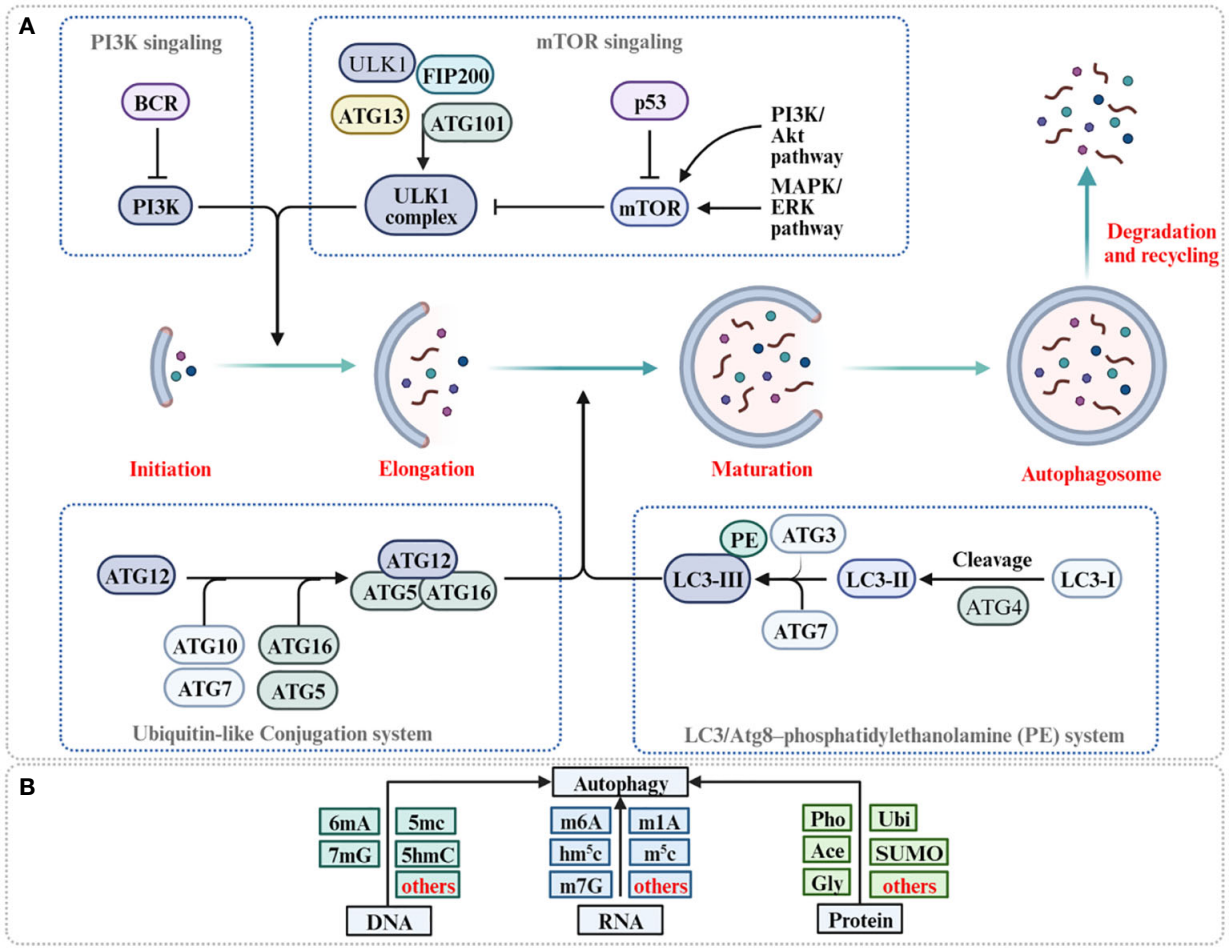
The process of autophagy and its regulation for 6mA methylation, m6A methylation and protein modification. **(A)** Autophagy is initiated with the formation of ULK1 complex (ULK1-ATG13-FIP200-ATG201) and PI3K complex facilitate the formation of phagophore. ATG5-ATG12-ATG16 complex and PE-conjugated-LC3II promote phagophore elongation and autophagosome formation. Autophagosome fusion with the lysosome results in the degradation of target molecules. **(B)** Methylation of DNA, methylation of RNA, and protein modification of protein are reported to participate in autophagy regulation (Pho, phosphorylation; Ace, acetylation; Ubi, ubiquitination; Gly, glycosylation).

Macroautophagy, henceforth referred to simply as autophagy, represents an evolutionary conserved cellular process, it not only serves as a pivotal mechanism for preserving intracellular balance by methodically dismantling and subsequently recycling cellular components, but also plays a dual role in cancer (20, 21). Recent studies have expanded our comprehension by illuminating the intricate relationship between autophagy and epigenetic modifications (22, 23). The core of autophagy is the formation of double-membraned structures called autophagosomes, proficiently encapsulating cytoplasmic components, including organelles and proteins, marked for degradation (24). Subsequently, these autophagosomes merge with lysosomes, culminating in the formation of autolysosomes, wherein the engulfed material succumbs to the catabolic prowess of lysosomal hydrolases (25, 26). The ensuing breakdown products are then carefully recycled back into the cytoplasm, providing crucial sustenance for energy production and macromolecular synthesis, thereby furnishing steadfast support to cellular function during periods of exigency (27) (**Figure 1A**). Autophagy plays a fundamental physiological role, particularly in the elimination of damaged proteins and organelles during periods of stress and aging (28). This process is instrumental in orchestrating organismal development, working in synergy with the adaptive immune system, ensuring energy balance, and maintaining rigorous protein and organelle quality control (29, 30). Recent findings also suggest that autophagy might intensify cancer malignancy by fostering metastatic behaviors (31). Moreover, autophagy is central to enhancing cellular resilience against diverse challenges, from nutrient scarcity and oxidative stress to pathogenic threats, reinforcing cellular strength and integrity (32–34). Recent investigations have elucidated the complex interplay in autophagy regulation, highlighting the nuanced contributions of DNA methylation variants, particularly 6mA, 7mG, and 5mC, in concert with RNA methylation types, including m6A, m1A, and m7G, and synergistic protein modifications (35–37) (**Figure 1B**). Collectively, these mechanisms ensure the fundamental principles of autophagy regulation, and more importantly, they shed light on the intricate role of autophagy in cancer.

In the early phases of tumorigenesis, autophagy functions as a protective barrier, eliminating damaged proteins and organelles. This crucial process prevents the accumulation of genetic abnormalities that could potentially trigger malignant transformations (38, 39). However, as tumors progress and mature, autophagy undergoes adaptations to support their growth in nutrient-deprived environments, assuming a dual role in cancer regulation (40). Research indicates that tumors, by harnessing autophagy’s ability to recycle nutrients, can sustain their growth, develop resistance to treatments, and even facilitate metastasis (41). This adaptability empowers tumors to thrive in challenging conditions, rendering them resilient targets for treatments (42). Moreover, the dual role of autophagy, both inhibiting and promoting tumors, adds complexity to therapeutic targeting (43). As cancer progresses, tumors exploit autophagy’s recycling abilities, evolving with heightened aggression, resisting treatments, and potentially spreading (44). Enhancing autophagy in mature tumors can bolster their resilience, suggesting that inhibiting autophagy could be a promising avenue for treatment. Although the precise regulatory mechanisms of m6A in eukaryotic mRNA and 6mA methylation in genomic DNA are not fully understood, the m6A modification, governed by the interplay of methyltransferases and demethylases, likely exerts an influence on mRNA behavior, potentially impacting crucial autophagic genes. Simultaneously, the presence of 6mA in genomic DNA introduces a distinctive epigenetic layer, influencing the transcription of autophagy-related genes (23).

Given autophagy’s dual influence on cancer, it presents both therapeutic challenges and opportunities. Viewing from a genomic lens, autophagy’s diverse roles in cancer unveil complex therapeutic avenues. The deep connection between genomic alterations and autophagy is not only crucial but warrants thorough exploration, as it might redefine our perspective and strategies in cancer therapy.

## 3 6mA methylation in autophagy regulation

6mA methylation, characterized by the methylation of adenine at its sixth position, has recently gained recognition as a significant epigenetic marker prevalent across the genomes of diverse organisms (45). DNA methylation, a crucial epigenetic mechanism, influences gene expression and various cellular processes without altering the DNA sequence itself (37). One prominent form of methylation is 5-methylcytosine (5mC), which is prevalent in mammals and recognized for its ability to suppress transcription factor-DNA interactions. Apart from 5mC, genomic DNA across different species showcases other methylated bases, such as 6mA and N4-methylcytosine (4mC) (46, 47). Contrary to 5mC, 6mA and 4mC are primarily recognized for their functionality in lower organisms like bacteria and protists (48). Most importantly, 6mA methylation assumes a pivotal role in DNA replication, repair, gene expression, and interactions with pathogens (49). Changes in 6mA methylation might contribute to the development of resistance to cancer therapies. Understanding these mechanisms could lead to new approaches to prevent or overcome resistance, such as combination therapies that include epigenetic drugs. In order to comprehend this indispensable role, we offer a general perspective on the mechanism of 6mA methylation, particularly, we focus on the interplay between 6mA and autophagy to further indicate the relationship between 6mA methylation, autophagy, and cancer.

### 3.1 Mechanism of 6mA methylation

6mA, a form of DNA methylation characterized the addition of a methyl group to the adenine base, is a well-documented epigenetic feature observed in bacteria and prokaryotes (50). While the presence of 6mA DNA methylation in plants and insects has been acknowledged for some time, its direct involvement in autophagy continues to be elucidated (51, 52). Intriguingly, roles for m6A in stem cell fate determination have also been discovered. For example, research has outlined a mechanism wherein m6A potentially modulates the stability of WUS and STM mRNAs, thereby influencing shoot stem cell fate determination (53). Moreover, scholarly attention has been focused on the 6mA demethylase that operates on dsDNA in eukaryotic organisms. This line of inquiry underscores the unique role of the CcTet D337F mutant protein as a crucial chemical biology tool for *in vivo* manipulation of 6mA methylation (54). Such discoveries highlight the distinct role of 6mA methylation and its prospective significance in orchestrating subsequent cellular processes. However, its existence and operational function within eukaryotes remain a contentious area within the ambit of scientific exploration. Abnormal patterns of 6mA methylation in cancer cells could serve as biomarkers for early detection, prognosis, or as indicators of therapeutic response. Identifying unique methylation patterns specific to cancer types or stages could lead to more precise diagnostic and prognostic tools. Contemporary research posits that 6mA might exert influence gene expression, transposon silencing, and environmental stress response.

**Figure 2:**
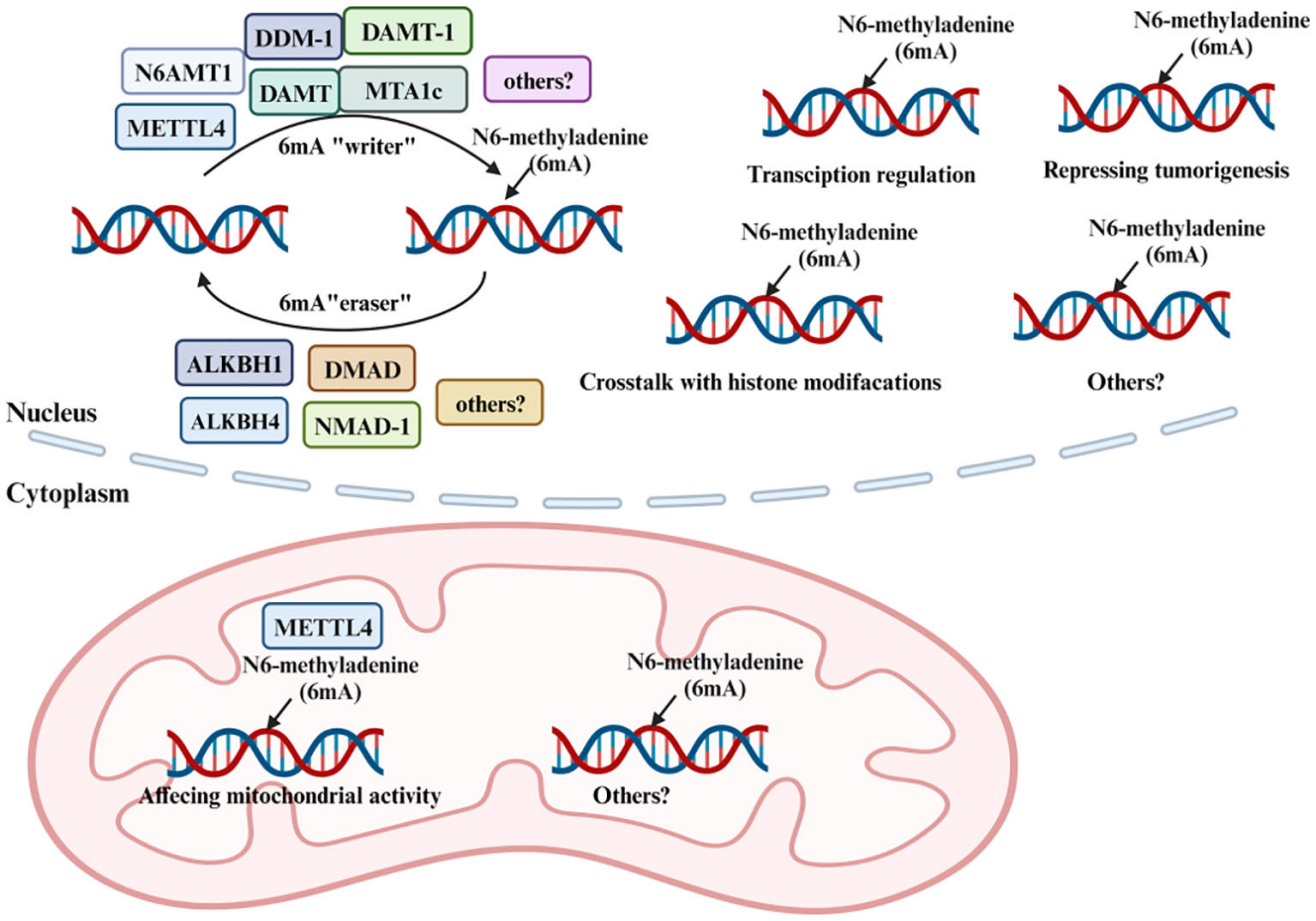
Methylation and demethylation of 6mA. 6mA methylation is a dynamic and reversible process coordinated by a series of methyltransferases (METTL4, N6AMT1, DDM-1, DAMT, DAMT-1, and MTA1c termed as “6mA writers”), demethylases (ALKBH1, ALKBH4, DMAD, and NMAD-1 termed as “6mA erasers”).

Enzymes such as N6AMT1, METTL4, DDM-1, DAMT, DAMT-1, MTA1c, METTL3, and METTL14 are postulated to be “writers” of this modification. In contrast, ALKBH1, DMAD, NMAD-1, and ALKBH4 function as “erasers”. N6AMT1, identified as the methyltransferase responsible for nuclear 6mA methylation, might potentially shape 6mA DNA patterns through an indirect mechanism (**Figure 2**) (55). Additionally, Jumu and SSBP1 are categorized as 6mA-DNA-binding factors and are considered “readers” (56, 57). Studies have shown that a decrease in N6AMT1 is correlated with reduced DNA 6mA levels, increased tumor progression, and an unfavorable prognosis for breast cancer (BC) patients. Specifically, silencing N6AMT1 using sh-RNA diminishes DNA 6mA levels and heightened proliferation and migration of BC cells. In contrast, overexpressing N6AMT1 produces the opposite effect (58). Conversely, METTL4, recognized as a member of the adenine methyltransferase clan, has showcased its proficiency in adding 6mA imprints onto DNA (59, 60). Furthermore, researches on METTL3 and METTL14, primarily acknowledged for their RNA m6A transcriptional finesse, has revealed their capability in imprinting 6mA onto DNA (61). In current research focusing on DNA repair, it has been observed that the involvement of METTL3 in regulating homologous recombination repair subsequently impacts the chemotherapeutic response in MCF-7 and MDA-MB-231 cells (62). Notably, METTL3 knockdown heightened the sensitivity of these BC cells to Adriamycin treatment, leading to a pronounced accumulation of DNA damage (63). According to research findings, the METTL3-METTL14 complex exhibits the potential to modify DNA, especially in contexts such as single-stranded DNA regions or areas with DNA damage (64). However, the specific role of METTL3 and METTL14 in 6mA DNA methylation remains unclear. It is imperative to conduct more in-depth research to conclusively determine the involvement of METTL3 and METTL14 in DNA methylation and to elucidate the functional implications of such modifications. ALKBH1, a distinguished member of the renowned AlkB protein family, serves as a pivotal DNA repair enzyme in eukaryotes and acts as a DNA demethylase by excising methyl groups from 6mA in DNA and m6A in RNA (65). Consistent with this, ALKBH4, shown to demethylate DNA 6mA, has the capability to inhibit 6mA methylation. Interestingly, research has provided evidence that the inactivation of BcMETTL4 leads to the downregulation of 13 genes with methylation within their promoter regions. Notably, the autophagy protein Apg6 in *B. cinerea* was distinctly identified as well (66). Concluding this investigation, the independent disruption of two genes, *BcFDH* and *BcMFS2*, while not directly studied within a mammalian context, could nonetheless illuminate potential parallels and offer novel insights into similar genetic mechanisms in mammals. Study have demonstrated that alterations in DNA methylation also play a pivotal role in tumorigenesis and tumor suppression, suggesting the complicated connection between 6mA methylation and autophagy regulation in cancer (67). The intricate interplay between methylation and demethylation processes, and their implications in cellular functions, especially in cancer, underscores the necessity for a deeper understanding of these mechanisms.

### 3.2 6mA methylation in autophagy and cancer

The intricate relationship between DNA 6mA and autophagy regulation in cancer has garnered significant attention in the field of cellular biology. As previously noted, the 6mA modification on DNA is prevalent across the genomes of both prokaryotes and eukaryotes. In prokaryotes, it plays a regulatory role in various functions, including DNA replication, repair, transcription, and bacterial resistance (68). In humans, 6mA methylation has been linked to several diseases. For instance, a notable example is the role of the ALKBH1-demethylated DNA N6-methyladenine modification, which triggers vascular calcification through osteogenic reprogramming in cases of chronic kidney disease (69). The role of autophagy in cancer is primarily influenced by various molecular regulators, including enzymes associated with 6mA regulation. While articles address the broader aspects of autophagy, one specific area of interest is the connection between enzymes regulating autophagy and 6mA modifications in the context of cancer. A comprehensive approach to understanding 6mA’s role in regulating autophagy would involve examining both its transcriptional regulation and its influence on autophagy-related proteins.

The role of 6mA in autophagy is a rapidly growing area of research, and understanding its precise function requires a comprehensive mapping of 6mA modifications across the genome. METTL4 is part of a subclade of MT-A70 adenine methyltransferases (70). Modifying the epigenetic landscape could potentially alter the immune response to tumors or change the behavior of surrounding stromal cells, which could be leveraged therapeutically. Recently, the human METTL4 protein was reported to demonstrate 6mA methyltransferase activity on mitochondrial DNA (mtDNA) (59). Due to its distinct methylation sites, akin to those of 5mC, the role of 6mA methylation in growth, development, and tumor progression becomes discernible. However, in contrast to 5mC methylation, 6mA methylation appears to demonstrate a reduced correlation with transcription, as observed in mitochondrial transcription *in vitro* (59). Instead, it exerts a more pronounced influence on cell survival, particularly due to its significant role in genes such as TBC1D3H, CSMD1, and ROBO2, which are characterized by unstable methylation sites (71). The presence of 6mA on the promoter regions of autophagy-related genes can influence their transcription. It has been reported that in glioblastoma, levels of both 6mA and the 6mA demethylase enzyme ALKBH1 are elevated (72). This raises the possibility that other yet-to-be-discovered DNA methyltransferases or demethylase might also contribute to the elevated levels of 6mA observed in glioblastoma. Notably, as recently reported, ALKBH4 impacts protein substrates, although its extensive role in DNA modification has yet to be revealed (73).

In a seminal investigation, researchers found that the nicotinamide nucleotide transhydrogenase (NNT) gene is silenced through DNA hypermethylation in the cisplatin-resistant A549 phenotype (A549/DDP). This discovery implies that elevating NNT expression in A549/DDP cells might counteract their natural resistance to cisplatin (74). Interestingly, the increased vulnerability of A549/DDP cells to cisplatin, facilitated by NNT, wasn’t mainly due to its traditional role in managing NADPH and ROS balances. Instead, it was largely attributed to NNT’s ability to inhibit protective autophagy in these cells. These detailed insights, derived from CRISPR-based DNA methylation editing, highlight a deeper, perhaps previously underestimated, link between DNA methylation and autophagy. Another insightful study revealed that, upon autophagy activation, the *de novo* DNA methyltransferase DNMT3A governs DNA methylation of MAP1LC3 loci, leading to a consistent reduction in MAP1LC3 isoforms transcriptionally. Fascinatingly, this reduced MAP1LC3 expression is evident *in vivo*, as seen in both zebrafish larvae and mice exposed to a brief autophagy stimulus (5). While these observations were particularly significant in zebrafish, a wealth of research has concurrently unveiled a profound association between DNMT3A and oncogenesis (75, 76). The expression profile of the DNMT3A protein in human tissue specimens and its potential susceptibility to DNMT inhibitors remains somewhat unclear, it’s noteworthy to mention that DNA hypomethylation-induced instability predominantly manifests a chromosomal character (77). In a study meticulously delineating the intricate structure and mechanism of DNMT3A protein’s, it was illuminated that the DNMT3A-DNA interplay—encompassing a target recognition domain, a catalytic loop, and a DNMT3A homodimeric interface—revealed that somatic mutations associated with hematological cancers within the substrate-binding residues not only attenuate DNMT3A activity but also precipitate CpG hypomethylation, thereby catalyzing the transformation of hematopoietic cells (78). As a result, understanding DNMT3A-driven methylation changes in autophagy, especially concerning cancer cell evolution, provides crucial insights for discussions on 6mA methylation and its ongoing research.

ALKBH1 belongs to the AlkB family of Fe(II)/α-ketoglutarate-dependent dioxygenases. It is adept at repairing methylation-related DNA damage through a distinctive oxidative demethylation process (79, 80). When the enzyme binds to a methylated base, its active site aligns with a Fe(II) ion and an α-ketoglutarate molecule. Subsequently, molecular oxygen is introduced and activated by the Fe(II) ion, which then targets the methyl group, transforming it into a hydroxymethyl group (81–83). This unstable group swiftly rearranges to form a transient hemiaminal intermediate, which decomposes to release formaldehyde, leaving behind the unmodified base. Simultaneously, the α-ketoglutarate undergoes decarboxylation, yielding succinate and carbon dioxide (84). This process readies the enzyme for future reactions. Through this intricate mechanism, enzymes like ALKBH1 play a crucial role in directly counteracting specific alkylation damages (85). This not only safeguards genomic integrity but also exert influence on gene expression. Elevated intracellular levels of αKG have been demonstrated to inhibit starvation-induced autophagy, suggesting a potential feedback mechanism during nutrient-rich conditions (86). Moreover, αKG derivatives, such as dimethyl α-ketoglutarate, have been shown to influence autophagic responses in specific pathological scenarios. Beyond its primary metabolic roles, αKG serves as a cofactor for dioxygenase enzymes involved in epigenetic modifications. This indirectly affects gene expression patterns associated with autophagy. Research has shown that DMKG, TFMKG, and O-KG elevate intracellular levels of α-ketoglutarate. This elevation, in turn, boosts baseline autophagy in cells cultured in complete medium (87). Such findings indicate that intracellular α-ketoglutarate can inhibit starvation-induced autophagy. Therefore, αKG plays an intricate role within the multifaceted autophagy network, functioning both as a metabolic regulator and an epigenetic influencer. This emphasizes its critical importance in maintaining cellular balance and highlights its potential therapeutic applications. The enzymatic activity of ALKBH1 heavily relies on the presence of αKG, which aids in the hydroxylation of the methyl group on alkylated bases, resulting in base recovery. The interconnected roles of αKG and ALKBH1 highlight their importance in upholding genomic integrity. This connection has potential ramifications in epigenetic alterations and gene regulation. Any disruption in αKG concentrations or ALKBH1 functionality can influence gene expression patterns, suggesting their wider significance in diverse cellular activities and pathological states. Furthermore, research has explicitly shown that the N6-methyladenine DNA demethylase ALKBH1 fosters gastric carcinogenesis by impeding NRF1’s binding capability. Notably, the 6mA sites are abundant in NRF1 binding sequences and are targeted by ALKBH1 for demethylation. The demethylation of 6mA by ALKBH1 hinders NRF1-mediated transcription of subsequent targets, including several genes in the AMP-activated protein kinase (AMPK) signaling pathway (88). Collectively, the intricate interplay between αKG, its derivatives, and ALKBH1 underscores the profound impact of these molecules on cellular processes, ranging from autophagy regulation to epigenetic modifications. The multifaceted roles of αKG, serving as both a metabolic pivot and an epigenetic mediator, along with ALKBH1’s crucial function in DNA repair and gene regulation, highlight the delicate balance that cells must maintain for optimal function. As we delve deeper into comprehending these molecular interactions, it becomes evident that they hold significant promise for therapeutic interventions, especially in the realm of genomic integrity and disease prevention.

After examining the existing frameworks, we suggest that high-resolution mapping could provide more profound insights into the distribution of 6mA modifications. This may illuminate specific genomic regions or genes affected by these modifications, which in turn play pivotal roles in autophagy regulation (89). Targeting 6mA methylation pathways could exploit synthetic lethality, where the combination of a genetic mutation in the tumor and the inhibition of a methylation pathway leads to cancer cell death but spares normal cells. As previously highlighted, the CRISPR/Cas9 genome-editing system emerges as a valuable tool for exploring the functional implications of 6mA modifications (90). Through CRISPR technology, genes linked to 6mA modifications can be either knocked out or overexpressed, providing researchers with a more comprehensive platform to observe the subsequent effects on autophagy. Additionally, the CRISPR system can be harnessed to either introduce or eliminate 6mA modifications at designated genomic locations, providing a straightforward approach to assess their functional significance (91). The identification and in-depth analysis of these enzymes are pivotal for understanding the molecular intricacies of 6mA regulation. Exploring these enzymes can also shed light on the temporal patterns of 6mA modifications, unveiling details about the timing and mechanisms through which these modifications impact autophagy.

## 4 m6A methylation in autophagy regulation

Methylation, including N1-methylation, 7-methylguanosine, 5-methylcytosine and N6-methyladenosine, involving the addition of a methyl group to a molecule and plays a crucial role in a myriad of biological processes, particularly in regulating the multifaceted functions of RNA (92–96). Similar to the mechanisms governing 6mA methylation, the orchestration of m6A modifications on RNA involves a tripartite regulatory system. Methyltransferases are responsible for the adding the methyl group, demethylases facilitate its removal, and specific binding proteins are responsible for its recognition. This coordinated modulation plays a crucial role in the precise regulation of autophagy processes (97). The m6A “writers”, which encompass METTL3, METTL4, METTL16, WTAP, RBM15/15B, VIRMA, and ZC3H13, are responsible for introducing the m6A modification onto RNA (98–100). These enzymes collaborate to identify specific RNA substrates and catalyze the transfer of a methyl group to the adenosine base. On the other hand, the m6A “readers” are proteins that recognize and bind to the m6A-modified RNA, thereby influencing RNA fate and function. The YTH domain family, which includes YTHDC1, YTHDC2, and YTHDF, constitutes a prominent group of m6A “readers” (101). Other notable m6A readers include Insulin-like Growth Factor 2 mRNA-Binding Proteins (IGF2BPs), the Heterogeneous Nuclear Ribonucleoproteins (HNRNP) family, and Eukaryotic Translation Initiation Factor 3 (eIF3), each of which play distinct roles in RNA metabolism, ranging from splicing to translation (102). Lastly, the m6A “erasers” encompass enzymes like FTO and the ALKBH family members ALKBH3 and ALKBH5, which remove the m6A modification, restoring the RNA to its unmodified state (103). Small molecule inhibitors that affect the activity of these proteins could modulate m6A levels and thereby impact cancer cell behavior. Together, these molecules form a sophisticated regulatory network that fine-tunes RNA function in response to cellular needs.

### 4.1 Mechanism of m6A methylation

**Figure 3:**
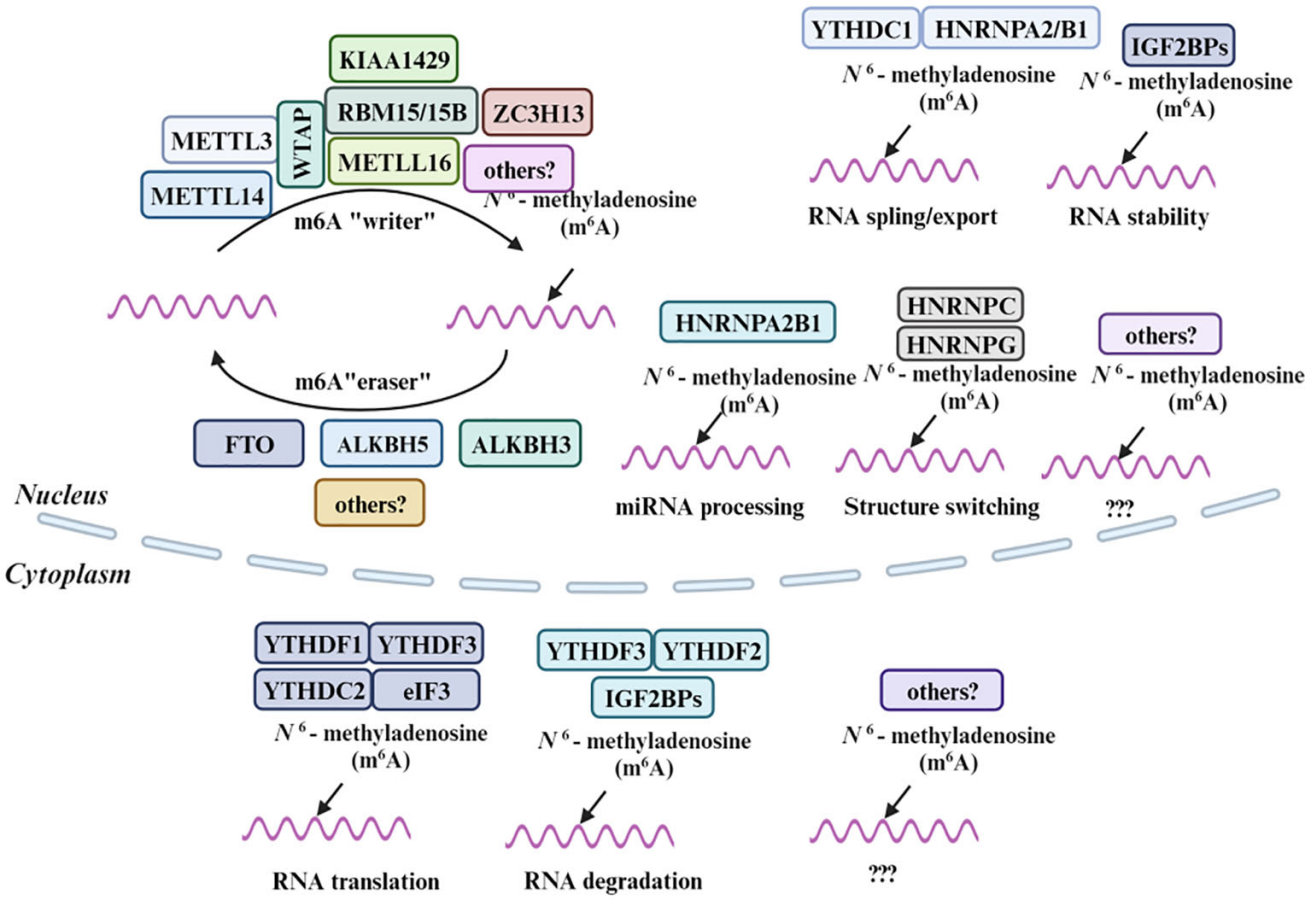
Molecular composition of m6A RNA methylation. M6A methylation is a dynamic and reversible process coordinated by a series of methyltransferases (METTL3/14, WTAP, RBM15/15B, METTL16, KIAA1429, ZC3H13 and termed as “m6A writers”), demethylases (FTO, ALKBH5 and ALKBH3 “m6A erasers”) and identifiers (YTHDF1/2/3, YTHDC1, HNRNPA2B1, HNRNPC, HNRNPG, eIF3, and IGF2BPs, “m6A Readers”).

Similar to 6mA methylation mechanism, m6A modification is also governed by a set of enzymes and binding proteins, including methyltransferases (“writers”), demethylases (“erasers”), and m6A-binding proteins (“readers”) (99, 104) (**Figure 3**). On one hand, the methyltransferases form the m6A methyltransferase complex, catalyzing the methylation reaction on adenine residues in RNA (105), on the other hand, the demethylases remove the methyl group from the N6 position of adenine in RNA, thereby reversing the methylation mark and restoring the unmodified state of RNA (106). Finally, the m6A marks recognized by m6A-binding proteins known as “readers”, play a crucial role in influencing the fate of the methylated RNA, such as regulating mRNA splicing, export, translation efficiency, and stability (107). In an analysis of EC samples, 17 m6A regulators, including YTHDC1, IGF2BP2, FTO, METTL14, and others, exhibited increased expression (108). Alterations in m6A modification have been implicated in the development of resistance to cancer therapies. By understanding these mechanisms, new strategies could be developed to prevent or overcome drug resistance. As research progresses in unraveling the mysteries surrounding these molecules, it paves the way for novel therapeutic strategies and a deeper understanding of the molecular underpinnings of cancer and autophagy.

The m6A “writers”, METTL3 initiating the m6A methylation process, while METTL14, another crucial component of the complex whose main function is to ensure the stability of the METTL3-METTL14 complex and facilitate substrate recognition, plays a central role in the transfer of the methyl group to the adenine base in RNA (109). It is noteworthy that the dysregulation of METTL3 and METTL14, and consequently m6A methylation, can have a significant impact on cancer progression. For instance, studies have identified that the Karyopherin Subunit Alpha 2, which is related to METTL3 and METTL14, plays a pivotal role in lung squamous cell carcinoma (110). In contrast to METTL3 and METTL14, METTL16 is responsible for methylating U6 spliceosomal RNA along with a subset of long non-coding RNAs and mRNAs (111). Furthermore, it was observed that METTL16 significantly amplify SOGA1 expression and mRNA stability, accomplished through its interaction with the “reader” protein, insulin-like growth factor 2 mRNA binding protein 1, which will be mentioned later (112). In addition, WTAP, a critical component of the m6A methyltransferase complex, plays an essential role in guiding the localization of the METTL3-METTL14 complex to specific nuclear sites, facilitating m6A methylation (113). Research has also identified WTAP’s association with a spectrum of immune cells in the tumor microenvironment, including cancer-related fibroblasts, myeloid dendritic cells, and various T cells (108). Notably, WTAP and m6A regulators like HNRNPC, YTHDC2, VIRMA, IGF2BP3, and HNRNPA2B1 showed up-regulation in both Esophageal squamous cell carcinoma and esophageal adenocarcinoma compared to normal samples. Furthermore, WTAP has been implicated in the regulation of autophagy in colon cancer cells, acting through the inhibition of FLNA via N6-methyladenosine (114). Specially, mRNA methylation also plays a pivotal role in autophagy of hepatoblastoma cell by enhancing LKB1, subsequently leading to increased phosphorylation of AMPK (115). Moreover, RBM15/15B and VIRMA are proteins that guide the m6A methyltransferase complex to specific sites in the m6A methylation process, ensuring precise and targeted m6A modifications (100). Additionally, ZC3H13, an integral part of the nuclear m6A methyltransferase complex, is essential for RNA methylation and also plays a role in facilitating the complex’s nuclear localization (116, 117).

The m6A “readers”, YTH domain family, which includes YTHDC1, YTHDC2, and YTHDF proteins, specifically recognize and bind to m6A marks on RNA molecules, allowing them to exert various regulatory functions, while the YTHDF proteins, including YTHDF1, YTHDF2, and YTHDF3, are involved in mRNA stability and translation (118–121). YTHDF1 has been distinctly recognized as a pivotal m6A reader protein responsible for BECN1 mRNA stability (122). The suppression of YTHDF1 can counteract the effects of BECN1 plasmid-induced HSC ferroptosis (123). This is further underscored by the role of YTHDF1 in enhancing BECN1 mRNA stability and activating autophagy, accomplished through its recognition of m6A binding site within the BECN1 coding regions (124). Similar to YTH domain family, eIF3 recognizes m6A marks and promotes the translation of m6A-modified mRNAs, thus influencing the efficiency of protein synthesis (125). Moreover, the IGF2BPs, including IGF2BP1, IGF2BP2, and IGF2BP3, constitute another family of m6A readers that recognize and bind to m6A marks and are involved in stabilizing their target mRNAs, protecting them from degradation (126). Members of the HNRNP family, such as HNRNPC and HNRNPG, recognize m6A marks and influence mRNA processing, also help interact with m6A-modified mRNAs, thereby affecting alternative splicing and transcript stability (127, 128). Research suggests that in prostate cancer, the elevation of circCSPP1, potentially catalyzed by HnRNP-L, triggers cellular autophagy via the circCSPP1-miR-520h-EGR1 axis, therefore, the HnRNP-L-regulated circCSPP1/miR-520h/EGR1 axis plays a pivotal role in modulating autophagy and advancing prostate cancer (129).

The “erasers” are FTO and ALKBH5, responsible for removing the m6A mark from RNA molecules (130–132). FTO, belongs to AlkB family, is capable of oxidatively demethylating m6A marks in RNA, converting m6A to adenosine. Consequently, it plays diverse roles in cellular processes, including energy homeostasis, regulating mRNA splicing, and influencing the timing of mitosis and meiosis while ALKBH3 and ALKBH5 exhibit a preference for demethylating m6A in single-stranded RNA regions, affecting mRNA export and metabolism, and also contribute to DNA repair processes (133, 134). Although the exact molecular mechanisms remain to be fully elucidated, emerging evidence suggests that ALKBH5 can influence the expression or activity of PHF20, DDIT4-AS1, and circCPSF6, thereby having an impact on autophagy (135–137). The intricate interplay between molecules like ALKBH5, PHF20, DDIT4-AS1, and circCPSF6 underscores the complexity of cellular processes and their implications in diseases such as cancer.

Absolutely, m6A methylation’s involvement in various aspects of RNA metabolism and its impact on different regions of RNA have profound implications for disease pathogenesis. The interplay between these writers, erasers, and readers ensures a finely-tuned and reversible m6A methylation process. Ongoing research in this field continues to illuminate the precise mechanisms and functional implications of m6A methylation in various biological processes and disease contexts.

### 4.2 m6A methylation in autophagy and cancer

As mentioned above, m6A plays a critical role in RNA metabolism, encompassing mRNA stability, translation efficiency, and splicing. It stands out as the most prevalent internal modification in eukaryotic messenger RNA and long non-coding RNAs. Moreover, it exerts widespread influence on diverse biological processes, including cell differentiation, tissue development, and stress responses. Particularly, aberrant m6A methylation patterns have been observed in various cancers (106).

METTL4, comparable in its functions to METTL3, is another established enzyme in RNA methylation, specifically involved in m7G methylation, which modulates RNA stability or structure. Consequently, METTL4 exerts an influence on the abundance of proteins essential for autophagy, which is also implicated in both the promotion and suppression of oncogenic pathways (123, 138). Furthermore, WTAP, a crucial component, interacts with METTL3 within the m6A methyltransferase complex, functioning in ensuring the proper localization and consequently implicates it in the regulation of m6A methylation (114). The Aberrant expression of WTAP has been observed in various cancers, including HCC (139). WTAP ensures the nuclear speckle localization of the METTL3-METTL14 heterodimer, thereby enhancing its catalytic activity (32). The significance of WTAP has underscroed by numerous studies, highlighting its pivotal role in a range of cancers, including those affecting liver, esophageal, breast, bladder, lung, and lymphoma system (140). In addition, insulin-like growth factor 2 mRNA-binding proteins are renowned for their role in binding and stabilizing target mRNAs, consequently promoting their translation (141). While their direct involvement in autophagy remains unclear, they theoretically could influence autophagy through their impact on mRNA stability and the translation of autophagy-related mRNAs (142). On the other hand, YTHDC1 is recognized for its capacity to bind m6A-modified RNA, exerting influence on various RNA processes such as splicing, export, and degradation (143). Recent studies have illuminated the multifaceted role of m6A modifications in determining mRNA fate, extending beyond traditional regulatory mechanisms. Specifically, m6A modifications have been demonstrated to facilitate the phase separation of their associated readers (144). A striking example of this is the modulation of cytosolic mRNA fate by m6A through its scaffolding function (145). While a direct association with autophagy has yet to be established, YTHDC1 could potentially impact autophagy by regulating the processing and stability of m6A-modified RNAs that encode proteins involved in autophagy (146).

Demethylases ALKBH3 and ALKBH5, both belonging to the AlkB family of Fe(II) and α-ketoglutarate-dependent dioxygenases, play pivotal roles as “erasers” in the context of RNA m6A modification. They have the potentially to influence the expression of autophagy-related genes through their impact on the RNA methylation (131, 147). Notably, ALKBH5 is not merely a passive marker; it actively modulates oncogenic processes, playing a crucial role in both the proliferation and invasion of cancer cells (148). In the context of gastric cancer, the expression of demethylase genes, FTO and ALKBH1, holds prognostic significance, possibly indicating their involvement in autophagy regulation (149). While an analysis of nine m6A-related genes revealed that elevated mRNA expression of FTO and ALKBH1 correlates with unfavorable overall survival in both the KM and TCGA cohorts, the TMA-IHC data contrasts this finding by showing a pronounced downregulation of FTO and ALKBH1 protein expression in gastric cancer tissues (150). A study revealed that the eIF3, central to protein synthesis initiation, can influence autophagy by controlling the translation of proteins in this process (151). Specifically, the eukaryotic translation initiation factor 3 subunit G (EIF3G) is implicated in the progression of human colorectal cancer (152). Functional assays revealed that EIF3G overexpression enhances HCT-116 cell proliferation, migration, and xenograft tumor growth, whereas xenograft tumors derived from EIF3G-silenced HCT116 cells exhibited reduced weights and volumes compared to those from control cells (151). However, the specifics of these potential interactions necessitate further elucidation. Similar to other complex members, VIRMA’s function in RNA methylation could potentially impact the stability or translational efficiency of autophagy-associated RNAs (100). Given that autophagy requires the intricate coordination of diverse RNAs and proteins, METTL16’s involvement in RNA methylation raises the possibility of it influence on autophagy, potentially through the methylation of RNAs implicated with autophagy-regulating proteins (112). In another study, it was reported that FTO can demethylate the mRNA of ULK1, a significant initiator of autophagy, leading to a decrease in ULK1 protein expression and hence suppressing autophagy (153). Specifically, FTO-driven autophagy, influenced by impaired m6A mRNA demethylation due to low-level arsenic exposure, promotes tumorigenesis and advances renal carcinoma via modulation of SIK2 mRNA stability (154). Concurrently, FTO enhances ovarian cancer cell growth by increasing proliferation, decreasing apoptosis, and activating autophagy (155). Additionally, it has been reported that on a mechanistic level, FTO functions as a tumor suppressor by regulating metastasis-associated protein 1 expression through an m6A-dependent pathway, which promotes metastasis in colorectal cancer under the hypoxia-mediated FTO downregulation (156). RNA-binding proteins RBM15 and its paralog RBM15B, integral components of the m6A methyltransferase complex, play a crucial role in RNA methylation. Targeting m6A regulatory proteins could be used in combination with other therapies to exploit synthetic lethality or to enhance the efficacy of existing treatments. While a direct correlation to autophagy is yet to be unequivocally established, their involvement in RNA methylation implies a potential role in modulating autophagy-associated RNAs, thereby influencing the proteins they encode (157).

The pivotal role of m6A methylation in cancer metabolism is evident in its influence on glucose, amino acid, and fatty acid metabolic processes. This influence extends further to impact metabolism-associated pathways and transcriptional regulators. The research delved into the phosphorylation and localization of YAP/TAZ, uncovering that m6A regulation of LATS1 affects the activity of the Hippo pathway in breast cancer cells (158). This underscores the significance of m6A regulation in modulating cell proliferation and glycolytic metabolism in breast cancer via the Hippo pathway component. 18 genes, including IGF2BP2, IGF2BP1, IGF2BP3, VIRMA, YTHDF1, YTHDF2, YTHDF3, ZC3H13, METTL14, ALKBH5, METTL3, RBMX, WTAP, YTHDC1, FTO, HNRNPC, HNRNPA2B1, and RBM15, were found to be verexpressed in HNSCC, indicating the close connection between m6A methylation and autophagy in cancer (159). Furthermore, another research revealed that METTL3-mediated m6A methylation of the mRNA encoding Transcription Factor EB, a principal orchestrator of lysosomal biogenesis and autophagy, enhances its translation (6).

In conclusion, akin to METTL3, METTL4, METTL16, WTAP, RBM15/15B, VIRMA, and ZC3H13, the potential influence of YTHDC1, YTHDC2, YTHDF, IGF2BPs, the HNRNP family, eIF3, FTO, and ALKBH3/5 on autophagy is intertwined with their roles in RNA metabolism, collectively regulating autophagy in cancer (**Table 1**). A deeper understanding of these relationships could provide valuable insights for innovative therapeutic strategies targeting conditions involving autophagy dysregulation.

**Table 1:** 6mA, m6A and autophagy associated factors involved in autophagy interaction ad their potential pathway in cancers.

6mA/m6A Enzymes	Type of cancer/cells	Up/Down	Pathway	Associated protein/factors
N6AMT1	BC (58)	Up	\	RB1, P21, REST, and TP53
ALKBH1	Glioblastoma (72)	Up	AMPK	\
DNMT3A	HSCs (75)	Up	\	DNMT3A1,DNMT3A2
METTL4	HepG2 (59)	Up	\	TFAM
METTL3	HCC (160)	Up	METTL3/FOXO3 axis	YTHDF1,FOXO3
	NSCLC (12)	Up	\	SQSTM1, LC3B-II
METTL14	PAAD (161)	Down	AMPKα, ERK1/2,mTOR	METTL3
	HCC (162)	Down	AMPKα, ERK1/2, mTOR	WTAP
METTL16	Colorectal cancer (112)	Up	METTL16/SOGA1/PDK4 axis	SOGA1, YY1, IGF2BP1
WTAP	HCC (115)	Up	p-AMPK	LKB1
	Colon cancer (114)	Up	WTAP/FLNA axis	FLNA
RMRP	NSCLC (163)	Up	TGFBR1/SMAD2/SMAD3	YBX1
	Glioma (164)	Up	RMRP/ZNRF3 axis, Wnt/β-catenin	IGF2BP3
ZC3H13	Cervical cancer (116)	Up	ZC3H13-CENPK axis	CENPK
	CRC (165)	Up	Ras-ERK	CENPK
IGF2BPs	NSCLC (166)	Up	TRIM25/circNDUFB2/IGF2BPs	circNDUFB2, TRIM25
eIF3	Prostate cancer (167)	Up	MAPK	circPDE5A
	OV (107)	Up	MAPK	YTHDF1
FTO	BC (168)	Up	FTO/miR-181b-3p/ARL5B	ARL5B
	OV (169)	Up	cAMP	/
ALKBH5	OV (148)	Up	EGFR-PIK3CA-AKT-mTOR	miR-7
	PAAD (170)	Up	PER1-ATM-CHK2-P53/CDC25C	BCL-2
	PAAD (159)	Up	ALKBH5-HDAC4-HIF1α	YTHDF2

HCC, hepatocellular carcinoma; NSCLC, non-small cell lung cancer; CRC, colorectal cancer; OV, Ovarian cancer; PAAD, Pancreatic Cancer.

## 5 Discussion

While the role of m6A in RNA metabolism has been extensively studied, the functions of 6mA, especially in relation to autophagy, remain largely unexplored. Current methodological approaches, despite their advancements, may not be refined to accurately delineate these epigenetic modifications. However, there is optimism in the scientific community, driven by the emergence of cutting-edge mapping techniques, sophisticated functional assays, and the revolutionary capabilities of tools such as CRISPR/Cas9 (91). How to find more methyltransferase and demethylases for 6mA need to worthy of in-depth study. The confluence of integrative omics paradigms, the granularity of single-cell analyses, and the predictive prowess of AI-driven methodologies heralds a renaissance in our comprehension (171). As researchers delve deeper into this field, several pertinent questions arise: How do external stimuli influence these methylation patterns and their subsequent impact on autophagy? Are there specific physiological contexts where these modifications exert a more dominant influence? Furthermore, can we strategically modulate these epigenetic markers for therapeutic purposes in diseases where autophagy is dysregulated? Most importantly, how do these modifications integrate into broader cellular processes such as homeostasis, growth, differentiation, and environmental responses? Are there specific cellular environments where 6mA plays an amplified role in regulating autophagy? how can the CRISPR technology be fine-tuned to elucidate the subtle roles of 6mA in autophagy and other cellular functions? What therapeutic potential lies in altering 6mA levels in diseases marked by autophagy anomalies? Does an intersection between DNA 6mA and RNA m6A in regulating autophagy for cancers warrant further study? In the intricate environment of tumor cells, the levels of 6mA and m6A methylation are in a state of constant flux. Gaining insights into whether one type of methylation can modulate another, and subsequently influence broader gene expression, holds significant potential. Are there innovative strategies we can employ to dynamically decipher these evolving mechanisms? Although the answers to these questions remain uncertain, they hold the potential to significantly reshape our understanding of cellular behavior and pave the way for new treatment options for numerous diseases.

Simultaneously, this intricate interplay between 6mA, m6A, and autophagy, while academically intriguing, could also pave the way for transformative therapeutic interventions (172), offering a unique perspective on autophagy in cancer. The potential of 6mA in DNA, particularly in plants like rice, has been investigated, shedding light on its role in various biological functions (173). On the other hand, the role of m6A in RNA metabolism and its potential impact on autophagy presents an intriguing prospect for therapeutic interventions, especially in conditions marked by autophagy dysregulation (174). Emerging research has also highlighted the potential of targeting the autophagy pathway for therapeutic interventions in diseases like COVID-19 (175). Furthermore, the response of the epigenetic machinery during viral infections, including the involvement of various transcription factors and epifactors, implies that targeting these pathways could provide alternative therapeutic strategies (176). These discoveries may contribute to a deeper understanding of the molecular mechanisms employed by 6mA and m6A-autophagy interaction in inducing human disorders.

In summary, the intricate dynamics of DNA and RNA methylation in relation to autophagy present a burgeoning frontier in scientific research. The potential of harnessing these epigenetic modifications for therapeutic purposes holds immense promise, though comprehensive inferences are constrained by limited findings. As our understanding of these molecular mechanisms, we stand on the cusp of a transformative era in medicine, potentially offering novel solutions for diseases that have previously defied effective treatment.

## Author contributions

LL: Formal analysis, Writing – original draft. YZ: Writing – original draft, Data curation. QZ: Writing – original draft. JZ: Writing – original draft. HL: Writing – review & editing. WW: Writing – review & editing, Funding acquisition.
